# Zinc Oxide Nanoparticles and Vitamin C Ameliorate Atrazine-Induced Hepatic Apoptosis in Rat via CYP450s/ROS Pathway and Immunomodulation

**DOI:** 10.1007/s12011-023-03587-2

**Published:** 2023-02-15

**Authors:** Eman T. Mohammed, Ghada M. Safwat, Esraa A. Bahnasawy, Abdel-Razik H. Abdel-Razik, Doaa Sh. Mohamed

**Affiliations:** 1 Department of Biochemistry, Faculty of Veterinary Medicine, Beni-Suef University, Beni Suef, 62512 Egypt; 2 Department of Histology, Faculty of Veterinary Medicine, Beni-Suef University, Beni Suef, 62511 Egypt

**Keywords:** Atrazine, Zinc oxide nanoparticles, Vitamin C, Hepatotoxicity, Cytochromes, Oxidative stress, Apoptosis

## Abstract

Atrazine, as an herbicide, is used widely worldwide. Because of its prolonged persistence in the environment and accumulation in the body, atrazine exposure is a potential threat to human health. The present study evaluated the possible protective effects of zinc oxide nanoparticles and vitamin C against atrazine-induced hepatotoxicity in rats. Atrazine administered to rats orally at a dose of 300 mg/kg for 21 days caused liver oxidative stress as it increased malondialdehyde (MDA) formation and decreased reduced glutathione (GSH) contents. Atrazine induced inflammation accompanied by apoptosis via upregulation of hepatic gene expression levels of NF-κB, TNF-α, BAX, and caspase-3 and downregulation of Bcl-2 gene expression levels. Additionally, it disturbed the metabolic activities of cytochrome P450 as it downregulated hepatic gene expression levels of CYP1A1, CYP1B1, CYP2E1. The liver function biomarkers were greatly affected upon atrazine administration, and the serum levels of AST and ALT were significantly increased, while BWG%, albumin, globulins, and total proteins levels were markedly decreased. As a result of the above-mentioned influences of atrazine, histopathological changes in liver tissue were recorded in our findings. The administration of zinc oxide nanoparticles or vitamin C orally at a dose of 10 mg/kg and 200 mg/kg, respectively, for 30 days prior and along with atrazine, could significantly ameliorate the oxidative stress, inflammation, and apoptosis induced by atrazine and regulated the hepatic cytochrome P450 activities. Furthermore, they improved liver function biomarkers and histopathology. In conclusion, our results revealed that zinc oxide nanoparticles and vitamin C supplementations could effectively protect against atrazine-induced hepatotoxicity.

## Introduction

The use of herbicides to control weeds has been a common practice in global agriculture in order to increase agricultural production. However, these chemical agents can represent a problem for human health [1]. Atrazine is a worldwide used herbicide that selectively inhibits photosynthesis in broadleaf and grassy weeds [2]. Although it has been banned in the European Union due to its high exposure potential for humans, it is still used in many countries [3]. Because atrazine (ATZ) is applied to agricultural land and contaminates surface and ground waters, atrazine exposure will occur. In addition, ATZ can remain for years after application [4]. Environmental and occupational exposure to ATZ can cause toxic effects on both human and animals. Atrazine is easily absorbed through the gastrointestinal tract. High concentrations of atrazine were found in the liver and kidneys of male mice. This bioaccumulation is probably due to the ability of the toxic substance to interact with the phospholipid components of biomembranes, preventing its excretion unless the substance undergoes a process that increases its solubility in water [5].

Recent studies have indicated that ATZ could cause oxidative stress by enhancing oxidation production, suppressing antioxidant enzyme activity, and ultimately activating apoptosis [3]. The liver is the first organ to be affected by ingested oxidants, and it also plays a crucial role in oxidative stress resistance. It is known that atrazine causes hepatic damage, DNA damage and genotoxicity in the stomach, kidney, and liver of rats, as well as oxidative stress in several experimental models [6]. Besides, atrazine also disrupts the regulation of cell cycle in hepatocytes in vitro [3], where it is undergoing biotransformation throughout phase I and phase II reactions. In recent years, it was found that cytochrome P450 (CYP450) could metabolize a wide variety of pesticides [7]. In mammals, numerous studies indicated that ATZ was metabolized primarily by CYP450. In order to interfere with CYP450 function, ATZ elevated CYP450 activity and CYP1 subfamily mRNA expression in common carps [8].

For balancing the extreme production of free radicals, the mechanisms of enhancing redox homeostasis should be maintained. Many natural compounds have been tested as antioxidants as part of these mechanisms [9]. Zinc is a necessary trace element for humans, animals, plants, and microbes. In recent decades, zinc has been widely considered to determine its physiological and biochemical functions. Additionally, zinc is a component of more than 300 different enzymes, transcription factors, and cell-signaling proteins that maintain the body’s normal immune functions, adjust its protein metabolism, maintain cell membrane integrity, and regulate cell differentiation and proliferation [10]. Zinc is a cofactor for various enzymes and is essential for many biological processes, such as controlling how cells respond to innate immunity and protecting against free radicals [11]. Zinc supplementation can be beneficial to prevent oxidative damage because it has been demonstrated that various cells and tissues exhibit oxidative stress under conditions of zinc shortage. As a cofactor of the antioxidant enzyme superoxide dismutase 1 (SOD1), zinc functions as a component of the cellular antioxidant defense system and shields cells from oxidative stress by promoting the biosynthesis of glutathione peroxidase (GPx), inducing the production of metallothioneins, and inhibiting NADPH oxidase [12]. Zinc exhibits an anti-inflammatory action in addition to its role in maintaining redox balance. Recent investigations have shown that zinc supplementation affects the NF-κB pathway via changing the activity of the zinc finger protein A20. The NF-κB pathway and the pro-inflammatory response appear to be negatively regulated by the zinc transporter SLC39A8 or ZIP8 [13].

Nanoparticles possess superior physical, chemical, biological, and thermal properties as well as better catalytic activities as compared to bulk materials [14]. Zinc oxide nanoparticles (ZnO-NPs) are considered the most utilized materials for biomedical applications with the ability to pass barriers causing efficient targeting of cells in many diseases [15]. ZnO-NPs have attracted tremendous interest in various fields, including anticancer, antibacterial, antioxidant, antidiabetic, and anti-inflammatory activities [16].

Ascorbic acid (vitamin C) is a water-soluble compound used as broadly as possible to include cosmetic, pharmaceutical, and agricultural fields because of its biological antioxidant property [17]. Low concentrations of ascorbic acid reduced reactive oxygen species (ROS) helping in maintaining the intracellular redox balance and minimizing the free radicals that caused cell damage. Also, it was documented that ascorbic acid could neutralize the ROS produced from the imbalance between intrinsic antioxidant and oxidative stress. Additionally, it decreases inflammation through decreasing C-reactive protein and pro-inflammatory cytokines [18]. Moreover, it has been proved to restore vitamin E antioxidant ability [19] and could counteract the harmful effects of several xenobiotics [20].

Therefore, the present study aimed to investigate the protective effects of both zinc oxide nanoparticles and ascorbic acid against atrazine-induced hepatotoxicity in rats.

## Materials and Methods

### Feed

The standard normal rat chow was acquired for feed manufacturing from the Mecca factory in Beni Suef Governorate, Egypt. According to the calculated chemical composition, it includes yellow maize, soybean (48%), calcium carbonate, dicalcium phosphate, sodium chloride, DL-methionine, L. lecithin, and gluten maize (60%). The chemical analysis of the standard normal chow diet showed not less than 21% crude protein, 2.8% crude fat, 55–70% carbohydrates, 2.4% crude fiber, 1–4% vitamin/mineral mixture, and metabolic energy 2950 kcal/kg. The composition of the feed ration during the experimental period was shown in Table 1.

**Table 1:** The chemical composition of the rat diet

Ingredients	Calculated chemical composition %
Crude protein	21%
Crude fat	2.8%
Carbohydrate	55–70%
Crude fiber	2.4%
Vitamins/mineral mixture	1–4%
Metabolic energy	2950 kcal/kg

### Chemicals and Reagents

Atrazine (2-chloro-4-ethylamine-6-isopropylamine-s -triazine, ATZ) with 80% purity and ascorbic acid were purchased from the International Company for Scientific and Medical Supplies, Cairo, Egypt. Zinc oxide was purchased from Sigma-Aldrich, Egypt. The commercial diagnostic kits used for assaying MDA and GSH were obtained from Biodiagnostic Company for Research Kits, Egypt. Total protein, albumin, ALT, and AST kits were obtained from Beta Lab group, Cairo, Egypt. All additional reagents were of analytical grade and were commercially accessible.

### Zinc Oxide Nanoparticle Preparation

High-energy ball mill (HEBM) method was used to manufacture ZnO-NPs in accordance with Gusev and Kurlov [21]. ZnO-NPs were prepared at the Nanotechnology Lab, Faculty of Postgraduate Studies for Advanced Sciences, Beni-Suef University, Beni Suef, Egypt.

### ZnO-NP Characterization

A high-resolution TEM electron microscope (model JEM-2100, JEOL Ltd., Tokyo, Japan) was used to characterize ZnO-NPs in order to determine their size and shape. Using a Zetasizer version 7.11 (serial number MAL1121994) (Malvern Instruments Ltd, Malvern, Worcestershire, UK), the size distribution and zeta potential of ZnO-NPs in solution were measured. XRD analysis was also performed by a PANalytical X-ray diffractometer (Empyrean).

### Animals and Treatments

Sixty male Wistar albino rats, weighing 120–200 g, were obtained from Helwan Farm of Laboratory Animals, Cairo, Egypt. Rats were kept in cages made of polypropylene in conventional laboratory settings and given water ad libitum and fed a standard pellet diet. All research procedures were carried out consistent with the guide for the care and use of laboratory animals as well as the Research Ethical Committee of Beni-Suef University’s Faculty of Veterinary Medicine with an approval no. of 022–307.

One week following acclimatization, the rats were randomly assigned into six groups of ten rats each:

1. Control group (C group): rats were orally given 0.5 ml of distilled water.
2. Atrazine group (ATZ group): oral dosage of a freshly prepared atrazine (300 mg/kg) dissolved in distilled water was given to rats starting at the 9th day of experiment and continued for 21 days [22]. ATZ dose was chosen as LD_50_/10 based on the oral LD_50_ dose of 3090 mg/kg [23, 24].
3. ZnO nanoparticle group (ZnO group): oral dosage of a freshly prepared ZnO-NPs (10 mg/kg) dissolved in distilled water was given to rats for 30 days [25].
4. Vitamin C group (Vit. C group): rats were orally administered freshly prepared ascorbic acid (200 mg/kg) dissolved in distilled water, for 30 days [26].
5. ATZ + ZnO nanoparticles group (ATZ + ZnO group): Rats were orally administered ATZ (300 mg/kg) for 21 days along with ZnO-NPs (10 mg/kg) for 30 days.
6. ATZ + vitamin C group (ATZ + Vit. C group): rats were orally administered ATZ (300 mg/kg) for 21 days along with ascorbic acid (200 mg/kg) for 30 days.

The rats were monitored for general behavior, poisoning symptoms, mortality, and weekly weight changes throughout the experiment.

### Determination of Body Weight Gain %

Throughout the experiment, the body weights of each animal were weekly recorded. The following formula was used to calculate body weight gain % using the method of Chapman et al. [27]:

$$Body\;weight\;gain\;\%=\frac{Final\;body\;weight-initial\;body\;weight}{Initial\;body\;weight}\times100$$

### Sampling and Tissue Preparations

Twenty-four hours following the last dose, blood samples were collected via retro-orbital bleeding. Blood samples were left for 30 min at 37 °C to clot. For serum separation, clotted blood samples were centrifuged at 3000 rpm for 15 min. The obtained sera were preserved at – 20 ºC till they were used. Then animals were euthanized by cervical dislocation. Liver tissues were collected and washed with physiological saline (NaCl 0.9%) and then divided into three parts. The first portion was fixed in neutral buffered formalin 10% for histopathological examination. The second portion (0.5 g) was homogenized with 5 mL phosphate-buffered saline using a homogenizer (Teflon Homogenizer, India). Using a high-speed cooling centrifuge, the tissue homogenate was centrifuged at 3000 rpm for 10 min at 4 °C. The supernatants were kept at –20 °C until the oxidative/antioxidant indices were determined. The third portion of liver tissue was kept at − 80 °C for the determination of molecular parameters.

### Estimation of Oxidant/Antioxidant Biomarkers

The liver homogenate was used for the measurements of MDA and GSH levels according to the methods described by Albro et al. [28] and Ellman [29], respectively.

### Determination of TNF-α and NF-κB, BAX, Bcl2, Caspase3, CYP1A1, CYP1B1, and CYP2E1 mRNA Expressions by Real-Time Polymerase Chain Reaction

Total RNA was isolated from the liver tissue according to the manufacturer’s instructions. The concentration of RNA was measured using a UV spectrophotometer. The extracted RNA was reverse transcribed into cDNA using high-capacity cDNA reverse transcription kit (#K1621, Fermentas, USA). Real-time qPCR amplification and analysis were performed using an Applied Biosystem with software version 3.1 (StepOne™, USA). The primers used in the amplification are shown in Table 2. The reaction contained SYBR Green Master Mix (Applied Biosystems). Data from real-time assays were calculated using Applied Biosystem software. To calculate the relative expression of the genes examined, the comparative threshold cycle approach was used. The glyceraldehyde-3-phosphate dehydrogenase (GAPDH) gene was used for the normalization of these data. All of these processes were carried out in agreement with the procedure defined by Livak and Schmittgen, [30].

**Table 2:** The primer sequences used for amplification of mRNAs encoding TNF-α and NF-κB, BAX, Bcl2, caspase3, CY1A1, CY1B1, and CY2E1 genes

mRNA	Sequences (5` → 3`)	Accession No
TNF-α	F: GCAGGACTTCTTCAGCGG ACATG R: GTTAGGTTCAGCTCGCCTCTTCAC	NM_012675
NF-κB	F: GTCTCAAACCAAACAGCCTCAC R: CAGTGTCTTCCTCGACATGGAT	NM_199267.2
BAX	F: CCGGCAGGCCCATACTGAAT R: CTTGGACAGGGCAGATAGCC	NM_001042451.2
BCL2	F: TGATAACCGGGAGATCGTGA R: TCGCCAACGCTGGGCCTGCG	NM_020008
CASPASE-3	F: GATCACAGCAAAAGGAGCAGT R: CTCCACTGTCTGTCTCAAT	NM_012922.2
Cyp1A1	F: GAGACAGTATTGTGTAGTCCAAGT R: CAAGAGACCAAGAGCTGGTGTA	NM-012540.2
Cyp1B1	F: GAGAGTTGGTGGCAGTGTTGGTG R: CTCGGCATCGTCGTTGGTTGTAC	NM_012940.2
Cyp2E1	F: GAATGGGGAAACAGGGTAATG R: CAGAAATGTGGGGTCAAAAGG	NM_031543.2
GAPDH	F: CACCCTGTTGCTGTAGCCATATTC R: GACATCAAGAAGGTGGTGAAGCAG	NM_017008

*GAPDH*, glyceraldehyde-3-phosphate dehydrogenase; *F*, forward; *R*, reverse; *TNF-α*, tumor necrosis-α; *NF-κB*, nuclear transcription factor; *Bcl-2*, B cell lymphoma-2; *BAX*, Bcl-2-associated X protein; *Cyp1A1*, Cyp1B1, Cyp2E1, cytochromes P450 (1A1, 1B1, 2E1)

### Determination of Liver Function Tests

The serum levels of both albumin and total proteins were estimated by spectrophotometer using commercially available kits according to the methods described by Doumas et al. [31] and Henry [32], respectively. Albumin was subtracted from total serum proteins to calculate the levels of serum globulins, and the albumin/globulin ratio (A/G ratio) was calculated using the equation *A/G ratio* = *albumin/globulins* [33]. The enzyme activities of both ALT and AST were determined according to Steven [34].

### Histopathological Examination

Liver samples were dissected from all studied rats of all groups, directly immersed in 10% neutral buffered formalin fixative for 48 h. The specimens were subjected to the routine histological technique by dehydration in ascending grades of ethyl alcohol, clearance in xylene, impregnation in soft paraffin, blocking in hard paraffin, and then cut by rotatory microtome to obtain histological slides. The obtained slides were stained with hematoxylin and eosin stain (H&E) as a general stain for demonstration of tissue damage, and periodic acid–Schiff technique (PAS) was carried out for the mucopolysaccharide demonstration. All fixatives and stains were performed as outlined by Bancroft et al. [35].

### Statistical Analysis

All results were statistically assessed by one-way analysis of variance (ANOVA) using SPSS software, version 22 (Chicago, USA) and then comparisons by using the Tukey post hoc test. The data were exhibited as a mean ± standard error of the mean (SE). The differences were considered statistically significant at *p* < 0.05.

## Results

### Characterization of ZnO-NPs

#### TEM Analysis

**Fig. 1:**
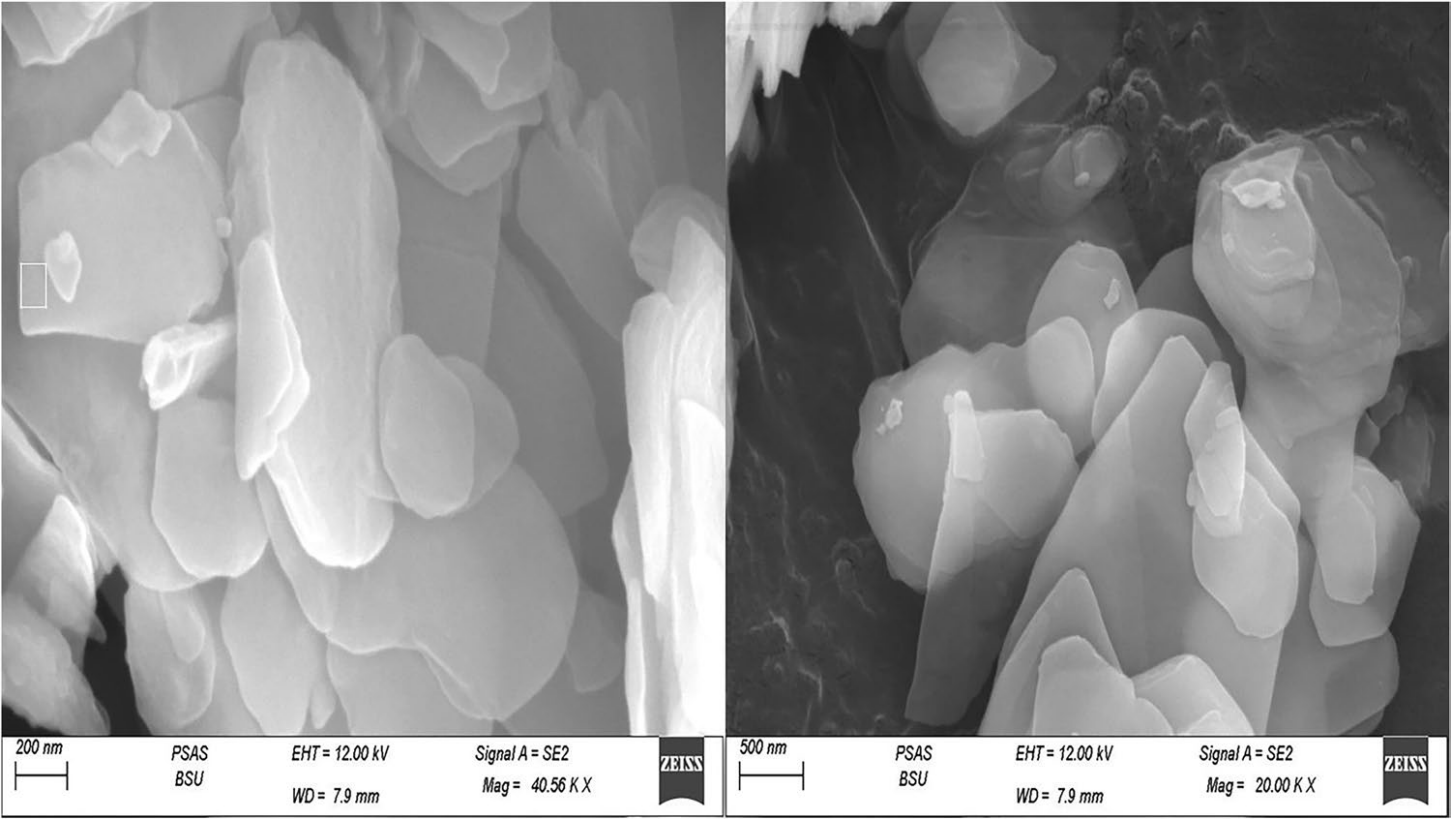
High-resolution transmission electron microscope (HRTEM) images of ZnO-NPs

The high-resolution transmission electron microscope (HRTEM) was used to verify the diameter, morphology, and dispersion of modified ZnO-NPs. Transmission electron microscopy (TEM) results were analyzed at 120 kV by the LEO system (model 912 AB) at the National Research Center, Dokki, Giza, Egypt. TEM image of modified ZnO-NPs was shown in Fig. 1. Results showed that NPs are uniformly distributed with no agglomeration, while the morphology of ZnO-NPs is the near-hexagonal shape and had a uniform nanometric size distribution which demonstrates the good quality of the ZnO-NPs. The size is in between 200 and 500 nm as the average size of nanoparticles.

#### Zetasizer and Zeta Potential Estimation

**Fig. 2:**
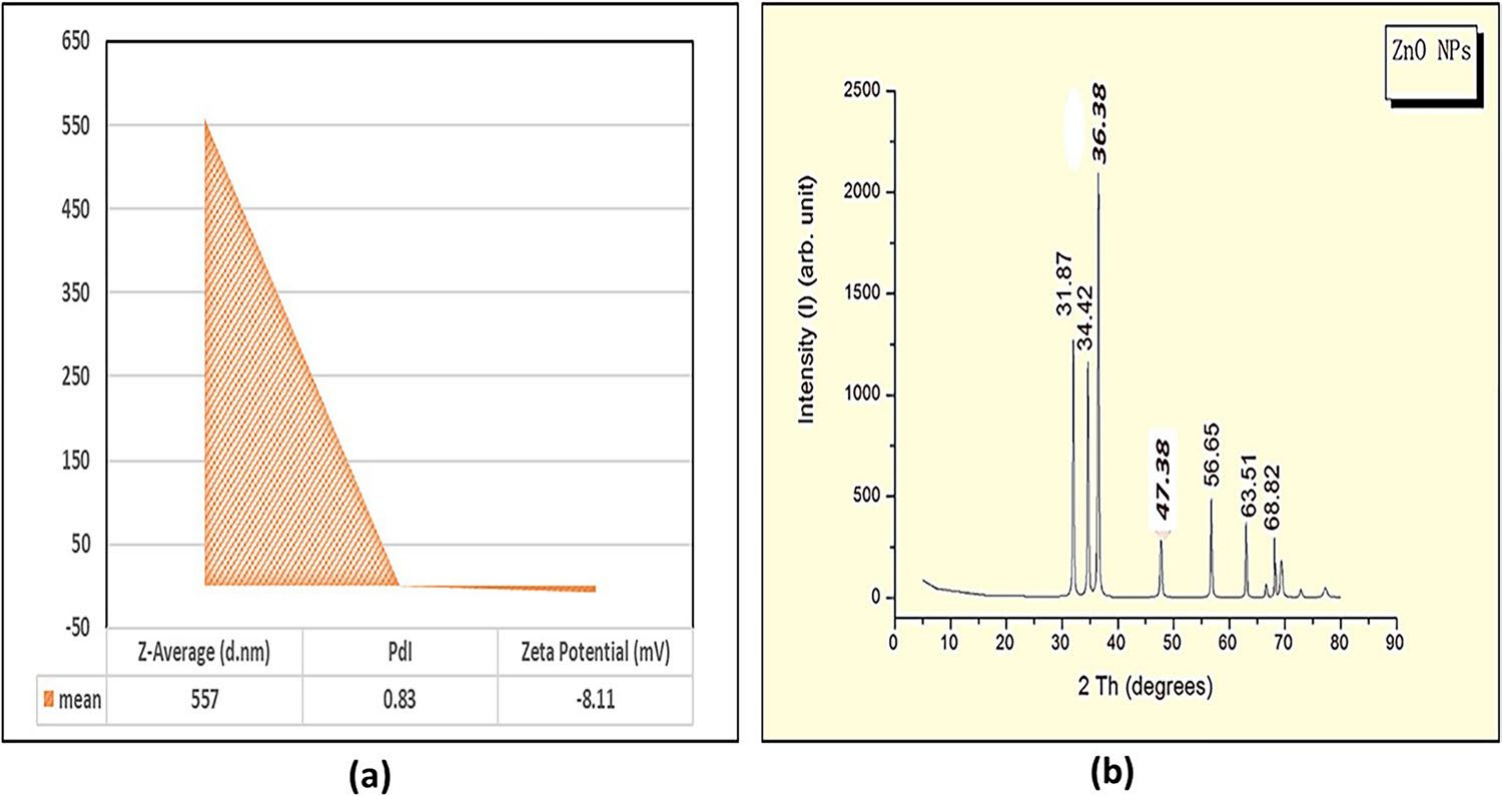
a Average size distribution and surface charge of prepared ZnO-NPs by using dynamic light scattering technique via Zetasizer instrument. **b** X-ray diffraction pattern (XRD) of ZnO-NPs

The average size distribution and the surface charge, as indicated by a Zetasizer version 7.11 (serial number MAL1121994) (Malvern Instruments Ltd, Malvern, Worcestershire, UK), were determined to be approximately 557 nm, and the apparent Zeta potential was approximately – 8.11 mV (Fig. 2a). The particles had a polydispersion index (PdI) of 0.83. The data obtained showed that the suspension of ZnO-NPs has good dispersion stability (Fig. 2a).

#### X-Ray Diffraction (XRD)

Figure 2b represents the X-ray diffraction pattern of ZnO nanopowder. PANalytical X-ray diffractometer (Empyrean) was used to perform XRD analysis using Cu Kα radiation (wavelength 0.120 nm) and found that ZnO nanoparticle is with space group P63mc. A definite line broadening of the XRD peaks indicates that the prepared material consists of particles in the nanoscale range. From this XRD pattern analysis, we determined peak intensity, position, width, and full-width at half-maximum (FWHM) data. The diffraction peaks located at 31.87°, 34.42°, 36.38°, 56.65°, 63.51°, and 68.82° have been keenly indexed as hexagonal wurtzite phase of ZnO with lattice constants *a* = *b* = 0.325 nm and *c* = 0.520 nm, and further, it also confirms the synthesized nanopowder was free of impurities as it does not contain any characteristics XRD peaks other than ZnO peaks.

XRD analysis of ZnO-NPs is shown in Fig. 2b. XRD profile of ZnO-NPs demonstrated nine pronounced diffraction peaks in the plane (100), (002), (101), (102), (110), (103), (200), (112), and (201), respectively, at the angle of 32°, 35°, 37°, 47°, 57°, 63°, 66°, 67.5°, and 70°. The obtained results indicate that the synthesized ZnO-NPs have polycrystalline wurtzite hexagonal shape without any effect on the phase of ZnO-NP crystallinity and in agreement to zincite (JCPDS 5–0664). There is no distinguishing peak of impurity which is suggesting that the synthesized ZnO-NPs were highly pure. Scherrer’s formula was used to estimate the mean crystallite size of ZnO-NPs [36, 37].

#### Clinical Signs and Body Weight Gain %

**Fig. 3:**
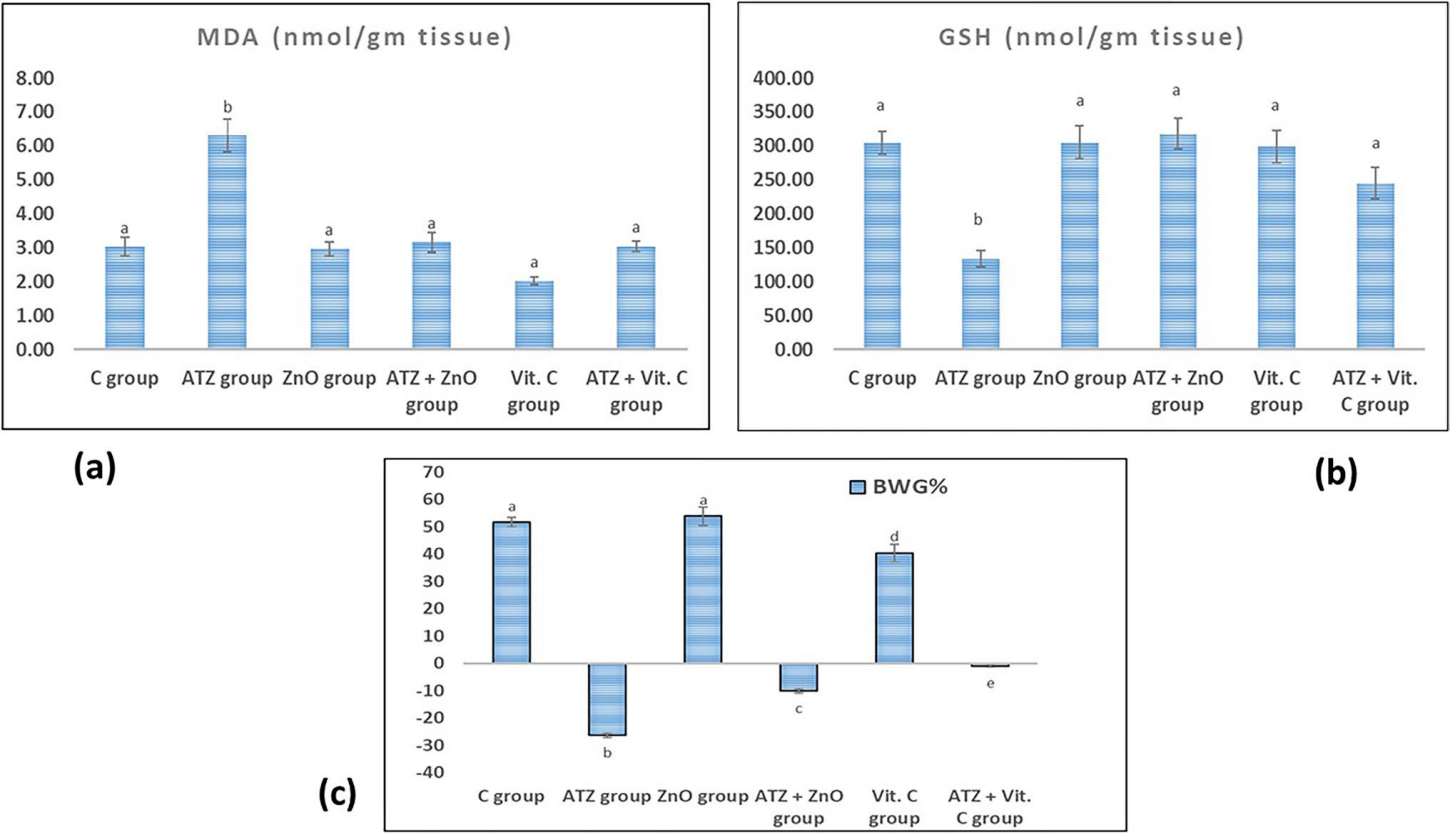
Changes in **a** hepatic MDA levels, **b** hepatic GSH contents, and **c** body weight gain % in different studied groups. The dissimilar superscript letters denote a remarkable difference at *P* value less than 0.05 between various groups

Throughout the experiment, there were no adverse clinical signs or death upon administration of ATZ or ZnO-NPs in rats. Atrazine induced a highly significant decrease in body weight gain % (BWG%) of rats in ATZ group compared with the control one, indicating its toxicity. The co-treatments with ZnO-NPs and ascorbic acid could significantly increase the BWG% in contrast to ATZ group (Fig. 3c).

### Effect of Atrazine, ZnO-NPs, and Ascorbic Acid on the Oxidant/Antioxidant Biomarkers in the Liver

Our results revealed that atrazine administration induced changes in oxidant/antioxidant indices as compared to the control group, while ZnO nanoparticles significantly modulated these changes as it decreased the hepatic MDA level and increased GSH content as shown in Fig. 3a and b. The antioxidant activity of vitamin C was noticed via the amelioration of changes in oxidant/antioxidant parameters induced by ATZ (Fig. 3a and b).

### Effect of Atrazine, ZnO-NPs, and Ascorbic Acid on the Pro-inflammatory Markers in the Liver

**Fig. 4:**
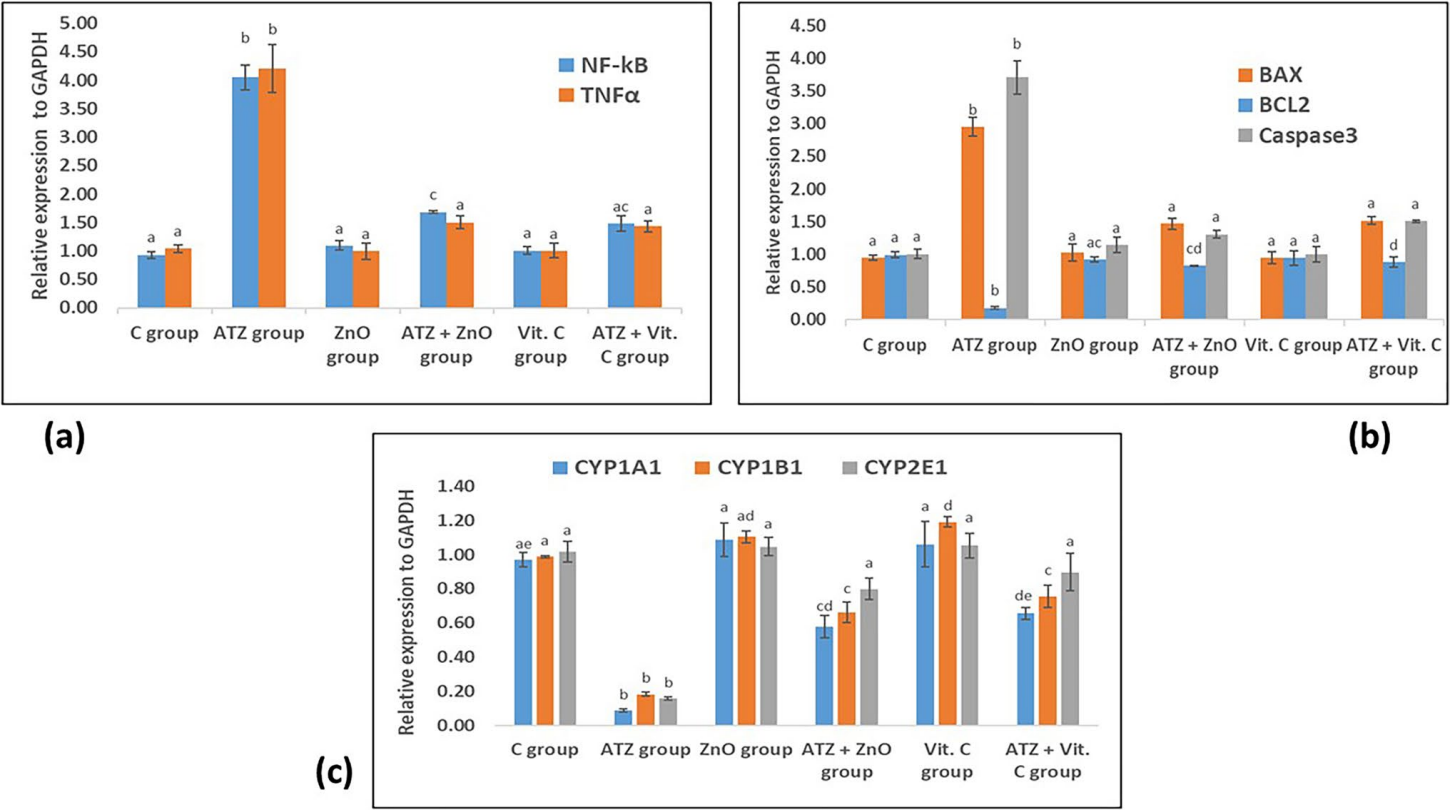
Changes in the hepatic gene expression levels of a NF-κB and TNF-α; b BCL2, BAX, and caspase 3; and **c** CYP1A1, CYP1B1, and CYP2E1 in different studied groups. The dissimilar superscript letters denote a remarkable difference at *P* value less than 0.05) between various groups

Data presented in Fig. 4a revealed a considerable increase in the gene expression levels of both tumor necrosis-α (TNF-α) and nuclear transcription factor (NF-κB) in the liver tissue of rats of ATZ group as compared to control group. On the other hand, the expression levels of these pro-inflammatory genes were significantly suppressed in both ATZ + ZnO-NPs and ATZ + vitamin C groups as compared to ATZ group.

### Effect of Atrazine, ZnO-NPs, and Ascorbic Acid on the Apoptotic Markers in the Liver

In this study, quantitative real-time PCR was used to analyze the mRNA expression levels of apoptotic markers (Bax and caspase-3) and anti-apoptotic protein Bcl-2 in hepatic tissues exposed to atrazine (Fig. 4b). It was noticed that atrazine significantly downregulated the gene expression level of BcL-2 gene and upregulated the expression levels of both BAX and caspase-3 genes in comparison with the control group. Meanwhile, the co-treatments with ZnO-NPs or vitamin C succeeded to regulate the expression levels of these genes as compared to ATZ group.

### Effect of Atrazine, ZnO-NPs, and Ascorbic Acid on CYP1A1, CYP1B1, and CYP2E1 Expression Levels in the Liver

The alterations in hepatic cytochromes were shown in Fig. 4c which revealed that ZnO-NPs group and vitamin C group showed a meaningful amelioration in the gene expression levels of CYP1A1, CYP1B1, and CYP2E1 which are downregulated in ATZ group.

### Effect of Atrazine, ZnO-NPs, and Ascorbic Acid on Liver Function Biomarkers

Liver function biomarkers presented in Table 3 showed a significant increase in the serum activity of ALT and AST enzymes accompanied by significant decreases in the serum levels of total proteins, albumin, globulins, and a significant increase in A/G ratio in the ATZ group in comparison with the control one. Additionally, co-treatments of ATZ-treated rats with ZnO-NPs or vitamin C could markedly improve the liver function as indicated by a restoration of these biomarkers toward the normal.

**Table 3:** Changes in liver function biomarkers of different studied groups

	Total proteins (g/dl)	Albumin (g/dl)	Globulins (g/dl)	A/G ratio	ALT (µ/l)	AST (µ/l)
Control	7.50 ± .12 ^a^	4.24 ± .08 ^a^	3.26 ± .14^a^	1.31 ± .07^a^	30.26 ± .04^a^	69.13 ± .72 ^a^
Atrazine	5.47 ± .25 ^b^	3.74 ± .03 ^b^	1.73 ± .14^b^	2.26 ± .22^b^	91.10 ± 4.35^b^	181.60 ± 15.22^b^
ZnO-NPs	7.50 ± .47 ^a^	4.22 ± .11^a^	3.28 ± .28^a^	1.33 ± .10^a^	32.80 ± 2.37^ac^	84.00 ± 3.55 ^a^
ZnO-NPs + atrazine	7.11 ± .08 ^a^	3.87 ± .12 ^b^	3.24 ± .15^a^	1.22 ± .10^a^	52.19 ± .53^d^	101.89 ± 4.09^a^
Vitamin C	7.55 ± .30 ^a^	4.20 ± .09 ^a^	3.35 ± .27^a^	1.29 ± .10^a^	41.16 ± .31 ^c^	85.29 ± 9.58 ^a^
Vitamin C + atrazine	7.07 ± .35 ^a^	3.89 ± .11^b^	3.18 ± .22^a^	1.26 ± .13^a^	62.30 ± 1.59^d^	139.70 ± 2.81 ^c^

Values are represented as mean ± standard error (*n* = 10). The different superscript letters mean a significant difference at *P* > *0.05* between different groups in the same column

### Effect of Atrazine, ZnO-NPs, and Ascorbic Acid on Histopathological Structure of the Liver

**Fig. 5:**
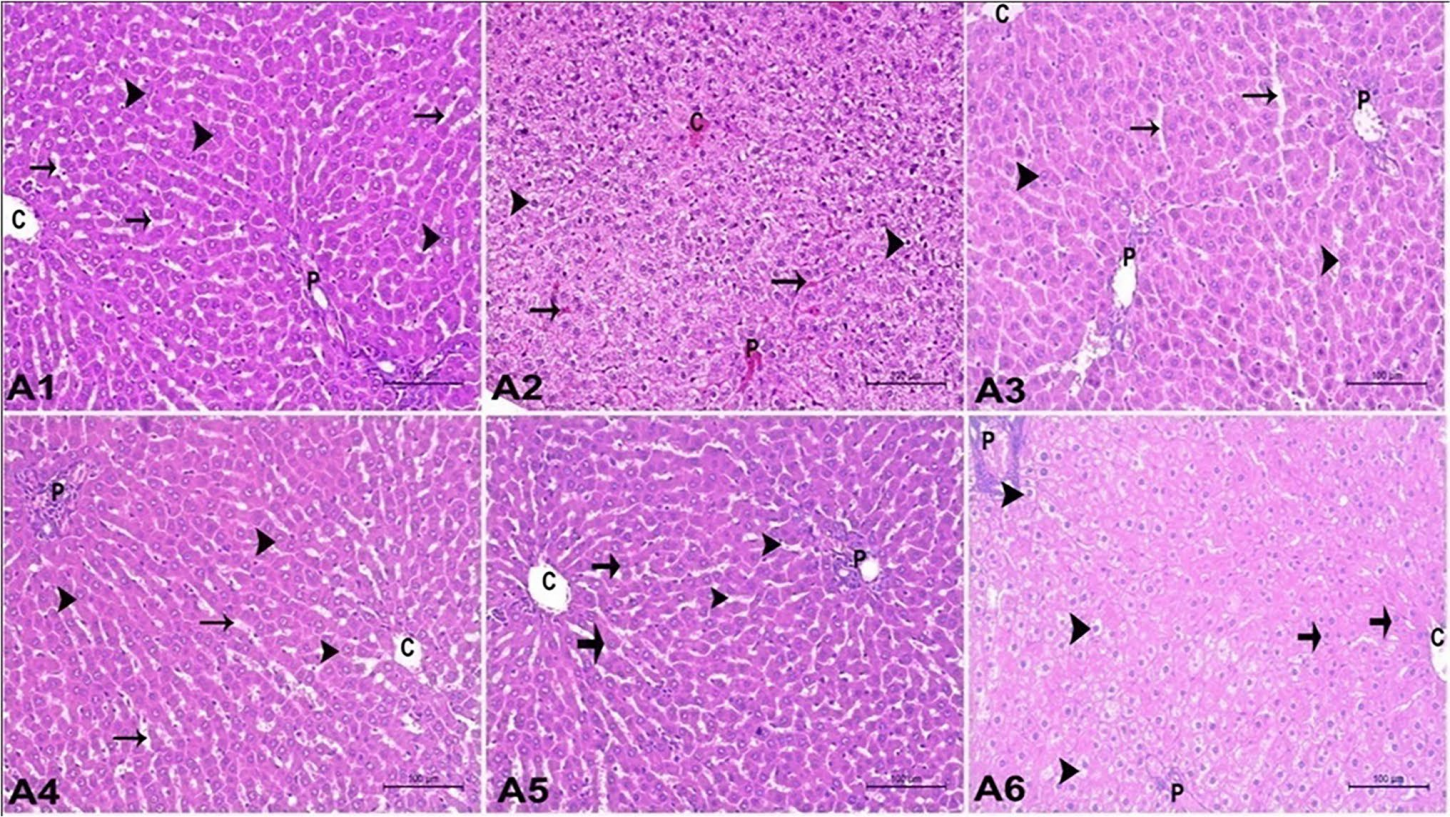
A photomicrograph of the liver in adult male albino rats showing the following: **A1** control group appeared as normal hepatic tissue architecture with central vein (C) and normal hepatocytes (arrow head) in hepatic cords separated by hepatic sinusoids (arrow) containing phagocytic cells as well as normal portal area (P). **A2** ATZ group showed disoriented hepatic with prominent portal area (P) suffered from congestion of portal blood vessels, proliferation of fibrous tissue. The hepatocytes (arrow head) appeared vacuolated and degenerated around dilated and congested blood sinusoids (arrow) and central vein (C). **A3** ZnO group showed normal hepatic tissue with normal hepatocytes (arrow head), blood sinusoids (arrow), central vein (c), and portal area (P). **A4** ATZ + ZnO group showed normal hepatic tissue with normal hepatocytes (arrow), blood sinusoids, central vein (c). The portal area (P) suffered from congestion of portal blood vessels. **A5** Vit. C group showed normal hepatic tissue with normal hepatocytes (arrow head), blood sinusoids (arrow), central vein (c), and portal area (P). **A6** ATZ + Vit. C group showed normal hepatic tissue with normal hepatocytes (arrow), blood sinusoids, and central vein (c), while few cells around the portal area suffered from degenerative changes and vacuolation (arrow head). The portal area (P) suffered from congestion of portal blood vessels. (H&E stain × 200)

**Fig. 6:**
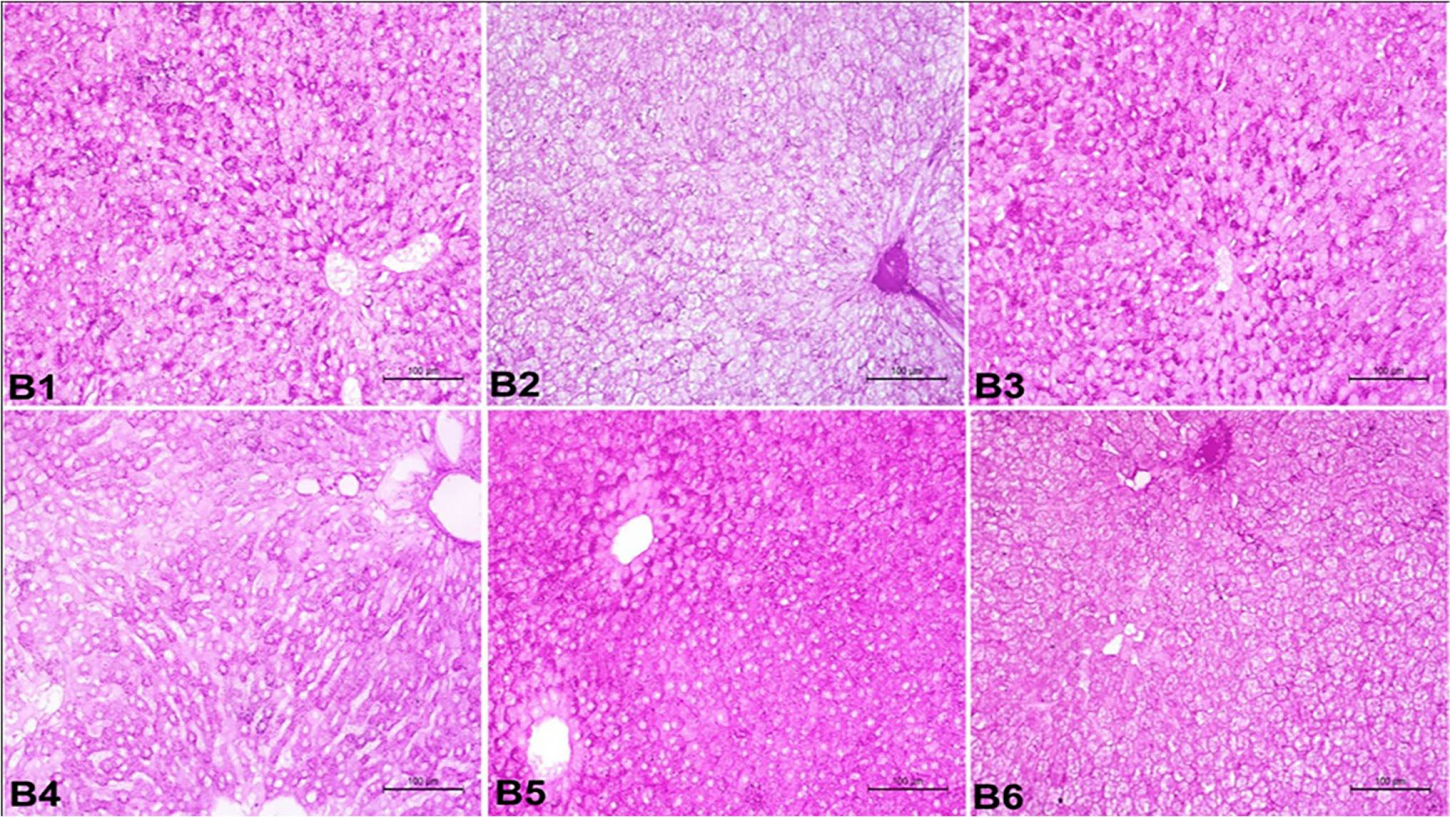
A photomicrograph of the liver in adult male albino rats (periodic acid–Schiff reaction, × 200) showing the following: **B1** control group showed strong reaction of PAS stain in the hepatocytes.** B2** ATZ group showed very weak reaction of PAS stain in the hepatocytes. **B3** ZnO group showed strong reaction of PAS stain in the hepatocytes. **B4** ATZ + ZnO group showed moderate to strong reaction of PAS stain in the hepatocytes. **B5** Vit. C group showed strong reaction of PAS stain in the hepatocytes. **B6** ATZ + Vit. C group showed weak to moderate reaction of PAS stain in the hepatocytes

Figure 5 showed a liver section stained by H&E and revealed that ATZ group showed disoriented hepatic tissue with a prominent portal area suffering from congestion of portal blood vessels and proliferation of fibrous tissue. The hepatocytes appeared degenerated and vacuolated around dilated and congested blood sinusoids and central vein. ATZ + ZnO-NP group and ATZ + Vit. C group showed normal hepatic tissue with normal hepatocytes, blood sinusoids, and central vein, while few cells around the portal area suffered from degenerative changes and vacuolation. Results of periodic acid–Schiff reaction were presented in Fig. 6 which showed that ATZ group displayed a very weak reaction of PAS stain in the hepatocytes, whereas ATZ + ZnO-NP group showed a moderate to strong reaction of PAS stain, and ATZ + Vit. C group showed weak to the moderate reaction of PAS stain in the hepatocytes.

## Discussion

This research is conducted to evaluate the possible protective effects of both ZnO-NPs and vitamin C against the adverse effect of atrazine on liver tissue in rats. ATR has the potential to cause oxidative stress, DNA damage [38], and pro-inflammatory and immunological impairments [39]. The change in oxidant/antioxidant status is frequently used as an indicator of the ongoing oxidative stress in a particular tissue. The lipid peroxidation process in an organism is initiated by free radicals. One of the byproducts of polyunsaturated fatty acid peroxidation in cells is malondialdehyde (MDA).

A rise in free radicals leads to an excess of MDA generation. A frequent indicator of oxidative stress and the antioxidant status of the cell is malondialdehyde level [40]. In the current study, we measured MDA and GSH levels as indicators for changes in oxidant antioxidant status. One important detoxification mechanism is chemical modification of the xenobiotic by covalent linkage to glutathione. Glutathione is a crucial endogenous tripeptide (g-glutamyl-cysteinyl-glycine) found mostly in the liver, kidney, and erythrocytes of mammals. It participates in a number of intracellular detoxification pathways. In both humans and rats, the cytosolic isoenzymes GST-T1 and GST-M1 exhibit genetic polymorphism, which defines a person’s hyper susceptibility to the harmful effects of xenobiotics. ROS overproduction impairs the intracellular GSH homeostasis, leading to GSH deficiency, a pathophysiological hallmark in alcoholic and non-alcoholic liver diseases. Judging on the basis of the evidence obtained from experimental research and previous clinical studies, GSH administration seems to be a promising strategy to recover oxidative stress-induced liver damages in alcoholic and non-alcoholic liver diseases [41].

In the present study, it was found that ATZ caused a significant increase in hepatic MDA level and a significant decrease in hepatic GSH level. These findings are in line with that of Adesiyan et al. [42] who documented that atrazine treatment impaired the antioxidant defense and increased lipid peroxidation levels in the liver. Jestadi et al. [43] demonstrated that ATZ causes oxidative damage through the generation of free radicals which attack cell membranes, causing their destabilization and disintegration.

Zinc oxide nanoparticles were used to modulate the potentials of oxidative stress in the tissues owing to the antioxidant properties and/or free radical scavenging capacity [44, 45]. Ascorbic acid is a water-soluble, non-enzymatic antioxidant that protects cells from ROS by directly interacting with lipid peroxides, increasing GSH levels and antioxidant enzyme activity, and preventing the subsequent apoptosis [46]. Concomitant with these facts, the current results indicated that the treatments with zinc oxide nanoparticles and ascorbic acid could significantly reduce lipid peroxidation and protein oxidation. This allowed the restoration of the hepatic MDA and GSH contents to normal levels (Fig. 3a and b).

According to the current study, oxidative stress initiated the pro-inflammatory cascades in hepatocytes as shown by the significant upregulation of both NF-κB and TNF-α expression levels in response to ATZ exposure (Fig. 4a). These findings are consistent with those of earlier studies that hypothesized that ATZ would have inflammatory and immune-suppressive effects. In addition, Abass et al. [47] and Xue-Nan et al. [48] claimed that ATZ might significantly enhance the levels of mRNA expression for NF-κB and the pro-inflammatory cytokine TNF-α. Furthermore, the increases in NF-κB activity and TNF-α expression levels caused by some pesticides, such as paraquat [49], were accompanied by an increase in ROS generation. It was reported that oxidative stress induced by atrazine acts as an initiator of cytokine release and cell damage [50]. ROS are well-known inducers of NF-κB [51] which regulates the transcription of numerous genes involved in inflammation, cell survival, and apoptosis through the initiation and regulation of the pro-inflammatory cytokines like TNF-α [52] which produced as a result of binding of NF-κB to a specific DNA response region in the nucleus [53].

The present results indicated that co-treatment of ATZ group with ZnO-NPs could decrease mRNA expression levels of NF-κB and TNF-α, which could be attributed to the anti-inflammatory activity of ZnO-NPs. These findings come in agreement with Mahmoud et al. [54]. Moreover, Kim and Jeong [55] revealed that ZnO-NP exposure inhibited the nuclear translocation of NF-κB by blocking IκBα phosphorylation and degradation and consequently decreasing the production of IL-1β and TNF-α in LPS-induced RAW macrophages.

Vitamin C administration significantly reduced the inflammatory reactions caused by ATZ by decreasing the hepatic expression of NF-κB and TNF-α (Fig. 4a). Accordingly, Radi et al. [20] found lower expression levels of hepatic TNF-α in abamectin and ascorbic acid-treated rats. Additionally, Bowie and O’Neill [56] first showed that millimolar doses of vitamin C inhibit multiple pathways to NF-kB through the prevention of IκB kinase enzyme complex (IKK) which in the case of TNF-α is dependent on the activation of p38 mitogen-activated protein kinase (P38-MAPK).

Recent studies have indicated that ATZ could cause oxidative stress through and ultimately activating apoptosis [57]. Our results revealed that ATZ administration induced apoptosis via the intrinsic pathway as it significantly downregulated the Bcl-2 gene expression level and upregulated the gene expression level of BAX in rat liver tissue. These findings come in agreement with Zaya et al. [58] who mentioned that ATZ could damage liver by increasing apoptosis rate and decreasing liver size in vivo. Furthermore, caspase-3 gene expression significantly increased in ATZ group. This was supported by comparable findings detected in the rabbit spleen by Morgan et al. [59].

The family of B cell lymphoma-2 (Bcl-2) proteins plays a pivotal role in the cellular regulation of apoptosis as it integrates pro- and anti-apoptotic signals within the cell [60]. The anti-apoptotic Bcl-2 and pro-apoptotic Bcl-2-associated X protein (Bax) are two members of the Bcl-2 family of proteins that regulate the intrinsic apoptotic pathway.

As shown in Fig. 4b, the significant decrease in hepatic Bax and caspase-3 and the concomitant increase in Bcl-2 expression levels induced by the co-treatment with ZnO-NPs are indications of the anti-apoptotic action of ZnO-NPs. In agreement with these findings, Barakat et al. [61] showed that the administration of ZnO-NPs significantly attenuated the elevated renal BAX triggered by cisplatin nephrotoxicity, and Awadalla et al. [45] showed that the TNF-α and apoptotic markers (caspase-3 and Bax) were expressed less in renal tissues after ZnO-NP administration.

According to the findings of our study, ascorbic acid protects hepatocytes against apoptosis by upregulating the expression of Bcl-2, which in turn inhibits BAX and stops the cascade of caspase activation, as indicated by the lower expression levels of BAX and caspase-3 in our study (Fig. 4b). These results are consistent with several recent studies that focused on the anti-apoptotic effects of ascorbic acid in ethanol-induced apoptosis [62] and in lung fibrotic damage caused by N-nitrosodimethylamine [63].

The liver is primarily responsible for the metabolism of toxic substances, and it is the major site of the cytochrome P450 (CYP) expression. Therefore, the effect of ATZ on liver-detoxifying enzymes is very important. CYP enzymes serve as terminal oxidases in the mixed-function oxidase system for metabolizing various endogenous substrates and xenobiotics including drugs and toxins [64]. The CYP family, such as CYP1A, plays an important role in the detoxification mechanism. The induction of hepatic CYP mRNA by certain classes of xenobiotics, including pesticides, has been suggested as an early warning system [65]. We examined the effect of atrazine on various CYP isoforms including CYP1A1, CYP1B1, and CYP2E1. Our results showed that the gene expression levels of CYP1A1, CYP1B1, and CYP2E1 were significantly downregulated in the liver tissue of ATZ-treated group in line with Salaberria et al. [66] who found a concomitant decrease in hepatic CYP levels with ATZ administration which alter hepatic metabolism and induce oxidative stress in vivo. The ability of a chemical to induce certain CYP isoforms is likely to be a critical factor in determining its toxic and carcinogenic potential, and generally, it is accepted that xenobiotics that induce CYPs are carcinogenic or toxic to humans [67]. The nuclear xenobiotic receptor response was found to be activated by ATZ, and the expression of many CYP isoforms, including CYP1A1, CYP1B1, and CYP2E1, was reported to be lowered [68].

Increasing interest has been focused on the alteration of the CYP contents and activities in inflammation and its related pathophysiological conditions. Our results showed that zinc oxide nanoparticles upregulated the expression of CYP1A1, CYP1B1, and CYP2E1 in line with prior findings of Goel et al. [69], who declared that co-administration of zinc to chlorpyrifos intoxicated animals normalized the enzymatic activities of cytochrome P450. He claimed that zinc is crucial in controlling the hepatic activity of drug-metabolizing enzymes. Our data revealed that ascorbic acid upregulated the expression of CYP disturbed by ATZ in agreement with Kim and Lee [70], who reported that vitamin C increased the CYP concentration and improved hepatic drug-metabolizing dysfunction, and this protection is, in major part, caused by decreased oxidant stress and lipid peroxidation.

Because the liver is typically the primary organ that ingested chemicals before reaching the body’s fluids, it is regularly exposed to high concentrations of these chemicals [71]. The above-mentioned oxidative stress and subsequent release of pro-inflammatory mediators and apoptosis induced by ATZ treatment explained the hepatic dysfunction accompanied by decreased BWG% and tissue injury biomarkers that we found in ATZ group. Our results revealed that atrazine administration significantly decreased BWG in line with previous data obtained by Simić et al. [72], while ZNO-NPs and vitamin C significantly alleviated these changes observed in body weight gain in agreement with Lang et al. [73] and Mamoun et al. [74], respectively. Results in the present study indicated a significant increase in serum levels of ALT and AST enzyme activities and a significant decrease in the serum level of total proteins, albumin, and globulins in the group exposed to atrazine (Table 3). These results come in agreement with that of Akhtar et al. [75] who declared that ATZ-exposed snow trout exhibited significant changes in ALT, AST, albumin, and total proteins. ALT is a cytosolic enzyme in liver and is regarded to be more selective for hepatic injury [5]. AST is a mitochondrial enzyme that is generally detected in plasma and is present in the heart, liver, skeletal muscle, and kidney [76]. The elevation of ALT activity in the present study was ascribed to the injury of hepatocytes initiated by atrazine [77], whereas the rise of AST is the result of apoptosis-triggered mitochondrial damage caused by ROS generated by atrazine [78].

All blood proteins, with the exception of gamma globulins, are produced in the liver [79]. Albumin and globulins were mostly responsible for the decline in total proteins as detected in the present study. This indicates that atrazine is cytotoxic to the liver and immune system. In this respect, similar results were reported for catfish [80], Nile tilapia [81], and mature male Japanese quail [82]. A/G ratio is significantly elevated in the atrazine group due to the marked hypoglobulinemia induced by atrazine toxicity in rats.

The co-treatments of ATZ-exposed rats with zinc oxide nanoparticles or ascorbic acid in our study modulated the alteration in ALT and AST enzymes and come in line with Mahmoud et al. [54] and Hamza et al. [83], respectively, which also restored the normal levels of total proteins and albumin as in agreement with Hassan et al. [84] and Abou-Kassem et al. [85], respectively, indicating their cytoprotective and antioxidant qualities. Previous studies have shown that vitamin C ameliorated the elevated serum ALT and AST activities in rat models of malathion hepatotoxicity [86] and organophosphate pesticide toxicity [87].

On the other hand, histopathological analysis revealed disoriented hepatic function with a notable portal area affected by congested portal blood vessels and proliferation of fibrous tissue. Around enlarged and congested blood sinusoids and the central vein, the hepatocytes seemed vacuolated and degenerated (Fig. 5 A2). Taken together, the results indicate that atrazine caused hepatotoxicity. These results were in line with those of Campos-Pereira et al. [5] and Batool et al. [88]. In addition, atrazine exposure caused a congestion of liver sinusoids and veins in quail [89] and frogs [90]. Liver fibrosis may be triggered by ROS-induced mitochondrial malfunction and weakening, which can cause cell death and amplify a pathway promoting fibrosis and collagen synthesis [91]. Periodic acid–Schiff (PAS) staining is a crucial technique for identifying carbohydrates and is frequently employed to assess the glycogen synthesis, storage, and hepatocyte function [92, 93]. In the present study, the hepatocytes showed a very weak reaction to PAS stain in ATZ group compared to the control one (Fig. 6 B2), indicating hepatotoxicity. Our work has shown that ZnO-NPs could inhibit rat liver fibrosis development as appeared normal hepatic tissue with normal hepatocytes which agrees with Bashandy et al. [94]. In addition, ZnO-NPs can control fibrosis through the lowering of hepatic lipid peroxidation which participates in fibrosis development [95]. On co-administration of vitamin C to ATZ-exposed group, there were significantly positive protective changes in functions and structure of liver. Vitamin C supplementation can cause a positive ameliorative effect against ATZ hepatotoxicity which was approved by Ozturk et al. [96].

## Conclusion

Based on the above data, atrazine induced hepatic dysfunction via affecting nuclear xenobiotic receptors and inducing apoptosis via activation of inflammatory mediators attributed to oxidative stress. Our result revealed that ZnO-NPs and vitamin C considerably exhibited antioxidant and anti-inflammatory activities and reduced apoptosis. Also, they regulated xenobiotic metabolizing enzyme activity. Consequently, ZnO-NPs and vitamin C could be recommended for protection against atrazine hepatotoxicity.

## Data Availability

The data used to support the findings of this study are available from the corresponding author upon request.
